# Estimating the Fiscal Value of Children Conceived from Assisted Reproduction Technology in Australia Applying a Public Economic Perspective

**DOI:** 10.36469/001c.133796

**Published:** 2025-04-18

**Authors:** Mark Connolly, Nikos Kotsopoulos, Jinjing Li, Georgina Chambers

**Affiliations:** 1 Global Market Access Solutions, Health Economics Unit, St-Prex, Switzerland; 2 University Medical Center Groningen, University of Groningen, Netherlands; 3 University of Athens, Greece; 4 Faculty of Business, Government and Law University of Canberra, Australia; 5 Faculty of Medicine and Health, Centre of Big Data Research in Health, Faculty of Medicine and Health University of New South Wales, Sydney, Australia

**Keywords:** assisted reproduction, pronatalism, in-vitro ferilization, public economics, infertility, financing health, fiscal analysis

## Abstract

**Background:** Public funding for assisted reproduction varies across countries, which can influence the numbers of infertile couples treated annually, and consequently the numbers of children born each year from this technology. As infertility is a medical condition treated within the healthcare system, it must compete against all other medical interventions for funding. This raises questions about how to evaluate a technology that gives rise to human life compared with other healthcare interventions that reduce morbidity and mortality. **Objective:** To evaluate annual public spending on assisted reproduction technology (ART) in Australia to determine the likely fiscal impact for government over the projected lifetime of an ART-conceived birth cohort. **Methods:** A public economic framework was used to evaluate the number of children born from ART procedures performed in Australia in 2021 based on projected future lifetime tax contributions and public benefits received. We leveraged data from the Survey of Income and Housing conducted by the Australian Bureau of Statistics and imputations from tax-transfer microsimulations over the lifetime of the cohort estimating cumulative net-taxes. Public spending per pupil for education and lifetime health costs (in Australian dollars) were included in the benefits estimates. **Results:** We estimated lifetime gross taxes per individual of A841 631,consistingofA580 182 in direct taxation of earnings and A261 448inconsumptiontaxes.AfterdeductinglifetimetransfersreceivedandARTtreatmentcosts,anART−conceivedchildwasprojectedtogenerateA70 688 in discounted lifetime net tax revenue. Based on average government spend per child, a lifetime fiscal benefit-cost ratio of 2.68 was observed. Based on the 2021 ART treatment cohort, the government was projected to net A$1.29 billion in future taxes over the lifetime of the 18 364 children born. **Conclusion:** A positive net fiscal gain was achieved from current government spending on ART. We observed that every A1spentonassistedreproductionyieldedA2.68 in future discounted net tax revenue. These findings were sensitive to economic conditions such as future wage growth and inflation.

## BACKGROUND

Treatment of infertility is unique among medical interventions in that it gives rise to the creation of human life. This is in contrast to the majority of medical interventions seeking to preserve life, prevent illnesses, or improve quality of life. Despite such uniqueness, infertility is recognized as a medical disease by global health authorities, including the World Health Organization, and treatments are delivered within the health sector and must compete for resources with all other available medical services.^1^ However, traditional health economic methods used to assess the cost-effectiveness of medical interventions by Health Technology Assessment agencies are not well suited for valuing a life not yet conceived, which is the case for fertility.^2^ Therefore, demonstrating the value to funders is challenging.^2^ Findings from the latest International Federation of Fertility Societies’ survey on global trends in reproductive policy and practice found that only 53% of countries reported some provisions for financial support for assisted reproductive technologies (ART) in their respective countries.^3,4^ Consequently, many people with infertility go without treatment and are unable to have children,^5^ causing personal suffering to the estimated 48 million couples, or 1 in 6 couples, affected by infertility globally.^6^

The funding of ART in Australia is supportive by international standards. The national insurance scheme, Medicare, reimburses all medically necessary ART treatments for its citizens with no funding limits based on female or male age, number of cycles undertaken, number of existing children, body mass index, or smoking. Both ART medical procedures and medication are covered, but patients must usually pay a co-payment for each cycle. The average cost of an ART cycle in Australia is estimated to be A$10 000-12 000, with Medicare reimbursing around 70% of this cost. As a result of the supportive funding arrangements, Australia has one of the highest per capita ART utilization rates in the world, with more than 5% of national births every year attributed to ART.^6–8^ This pattern is consistent with previous studies showing that greater funding of ART can positively influence national fertility rates,^9–11^ particularly influencing birth rates in older women and reducing rates of childlessness.^12,13^

Over the past few decades, Australia’s policy has leaned toward pronatalism to support and encourage family-building in the face of aging populations and the need to finance generous public benefits. Indeed, Australia’s “economic dependency ratio,” which measures the number of people aged 65 and over relative to those of any age who are employed, has increased from 33.2% in 2022-2023 to 45.4% in 2062-2063.^14^ While the funding for medically assisted reproduction is not explicitly done with pronatalist intent, the funding for infertility treatment does achieve the aims by which pronatalist policies are measured (ie, live births).^15^ The total fertility rate in Australia has fluctuated between 1.6 and 2 over the last 2 decades, reaching its lowest level of 1.58 in 2020.^16^ Pronatalist policies funded by the Australian government to support fertility include financial transfers, parental leave, and childcare. Australia has relatively generous, means-tested financial transfers in the form of Family Tax Benefits A and B, which reduce the costs of children to parents, based on the number of children, the ages of the children, and the family income. Australia has had paid parental leave for the primary carer, usually the mother, since 2011, and paid paternity leave since 2013. Childcare is primarily market based and is subsidized by the government through the Child Care Subsidy Policy.

Economists have long recognized that births and the rearing of children create economic externalities extending beyond the family unit.^17,18^ From the perspective of government and public finances, human capital and the size of birth cohorts can influence government spending, tax revenue, and tax rates.^19,20^ Knowing this enables one to evaluate the fiscal impact of policies that impact birth rates or workforce participation by quantifying future taxes at the individual and aggregate level relative to the costs of implementing any new policy. To inform the formulation of policies aiming to increase access to ART for infertile couples, we applied the above fiscal analytic approach in Australia to estimate the benefits for the Australian government from a single ART birth cohort based on investment costs paid by government in a single year.^21,22^

## METHODS

A “government perspective” public economic modeling framework was developed to evaluate annual Commonwealth Government spending on infertility care in 2021 in relation to the future lifetime discounted net taxes paid by the corresponding ART cohort of children born as a result of the Australian government insurance scheme. The analysis projects the fiscal life course of the ART-conceived children using a discounted cash flow approach to estimate the fiscal rate of return for government from public funding to fertility clinics.^22,23^ The analysis described here is consistent with the generational accounting framework used by governments around the world, including use of microsimulation modeling by the Australian government to project the fiscal consequences of future government policies and spending.⁠^19,24^

### Model Design

Longitudinal projections were made for the cohort of 18 514 ART-conceived children born in Australia in 2021 obtained from the Australian and New Zealand Assisted Reproduction Database (ANZARD).^25^ The reported ANZARD births have been adjusted for stillborn births and annual mortality adjustments using Australian life tables for 2019-2022 up to age 100 and applied to all calculations in the model.^26^ All calculations were performed in Microsoft Excel.

The framework is based on the methodology of generational accounting used by governments to evaluate lifetime tax burdens across generations based on social benefit promises and who will be required to pay for them.^19^ The generational accounting methodology is used to estimate the long-term fiscal impact of policy changes on government accounts, in which demography is the driving force that influences government costs; therefore, it is suitable for estimating the impact of funding fertility programs.

### Income Taxes and Disposable Income per Individual

Annualized statistics on age-specific direct taxes paid were obtained from the Survey of Income and Housing collected by the Australian Bureau of Statistics in the 2019-2020 financial year and imputed from STINMOD+, a comprehensive tax-transfer microsimulation model designed specifically for the Australian context.^27,28^ The taxes paid include income taxes, Medicare Levy, and Medicare Levy surcharge contributions. This provides a comprehensive overview of how potential ART interventions affect government welfare schemes at the federal level through future births (**Supplemental Table S1**). Estimates of indirect taxes paid by age are derived from individual disposable income, estimated from individual circumstances reported in the Survey of Income and Housing. Disposable income calculations include transfers from government, including transfers on behalf of children, allocated to parents. The disposable income was applied to the Australian Goods and Service Tax rate of 10% to approximate indirect tax revenue.^29^ Age-specific data are reported in **Supplemental Table S1**.

### Government Transfer Payments

Data on age-specific taxes and transfers were obtained from the Australian Tax and Transfer System. Specifically, the estimations are derived from the Survey of Income and Housing collected by the Australian Bureau of Statistics in the 2019-2020 financial year.^27^ All figures mentioned in the report are annualized and denominated in Australian dollars (A$) and pertain to the 2019-2020 financial year. The spending programs include family allowances and pension payments in older age groups. A methodological choice within this analysis is the allocation of child-oriented transfers, such as the Family Tax Benefit and Childcare Subsidy, directly to the children. Although these transfers are traditionally received by guardians, attributing them to the children allows the assessment of age-specific impacts on tax and transfers of funding policies. This facilitates a better understanding of welfare needs over the life course.

We accounted for future government commitments to provide health care to all Australians. We applied government estimates on age-specific health expenditure annually^30^ and adjusted for healthcare inflation. The age-specific health spending on which future projections were made is presented in **Supplemental Figure S1**.

The analysis captures the additional costs associated with educating a child born in Australia. The government educational spending per student was applied to determine age-specific costs after adjusting for the proportion of children attaining each level of education and adjusted for inflation over the years of schooling.^31^ The analysis does not include costs paid directly by individuals and nongovernment payers (ie, scholarships)

### Macroeconomic Input Parameters

To estimate the gross and fiscal transactions over the lifetime of a child requires factoring in macroeconomic parameters known to influence the individual input parameters and forecasts described here. The model applies macroeconomic parameters to wages and government costs to account for economic growth, which is standard practice in governmental policy evaluations as recommended by treasury departments.^32,33^ The time frame of the model was set to the age of 100 years to capture the majority of costs and transfers over the lifetime of a newborn. We applied a 2.7% wage growth pattern to age-specific earnings based on data reported by the Australian Bureau of Statistics over the period 2010-2021 and estimating the geometric mean.33 Cash transfer payments over the lifetime were inflated using the consumer price index during the period 2013-2019 (excluding pandemic period) based on data reported by the Australian Bureau of Statistics.^34^

### Assisted Reproduction Investment Costs to Australian Government

The investment case for ART in Australia was evaluated by applying the aggregate government spending on fertility services in 2021. Pharmaceutical costs were obtained from the Pharmaceutical Benefits Schedule Item Reports for Section 100 IVF medication, totaling A$151 571 261 in 2021.^35^ For the same year, Medicare benefits of A$333 123 150 were obtained from the Medicare Benefits Schedule for medical and laboratory services in relation to delivery of ART services,^36^ based on the annual number of births in 2021 (excluding stillborn births).^25^ Based on the reported aggregate government spending relative to the number of children born in 2021, we estimated an average cost per child of A$26 394 (aggregate spending ÷ number of ART-conceived children).^25^ As a strictly governmental perspective analysis, the costs paid by couples, or nongovernmental organizations on behalf of couples, are not included in the fiscal analysis. All costs are shown in 2021 Australian dollars (A$).

### Calculating Lifetime Net Tax Revenues

To reflect the net present value of investment in ART, we depreciated treatment costs attributable to an ART-conceived child over the lifetime of the child ($K_{o}$). Furthermore, in each year of life we derived age-specific net tax revenues from the government perspective based on government transfers and gross tax receipts and discounted these costs to the base year (**Equation 1**). For comparison, a similar calculation was performed for a naturally conceived child excluding ART costs:

(1)NPV=∑t=0T(Rt−Et(1+r)t)−Ko

where

Rt = Sum gross tax revenues accruing from the individual age t

Et = Sum government expenditures on the individual age t

R = Discount rate

T = Life expectancy

$K_{o}$ = Cost per live birth

The model estimates cumulative gross and net taxes transacted over every year of life for the ART-conceived child. The net fiscal benefit is considered the outcome of interest, as it demonstrates the net fiscal position at any stage of life. In addition, we estimate the fiscal benefit-cost ratio (fBCR), which reflects the lifetime net taxes paid in relation to the costs of conception and is derived by dividing the net fiscal return by the investment cost per live birth.

### Sensitivity Analysis

A 1-way sensitivity analysis was performed to explore the influence of key model inputs on model output. We focused the sensitivity analysis on those parameters which would have long-term impact on fiscal metrics and excluded short-term costs such as service delivery to couples with infertility, as this occurs in year 0.

## RESULTS

**Figure 1.**
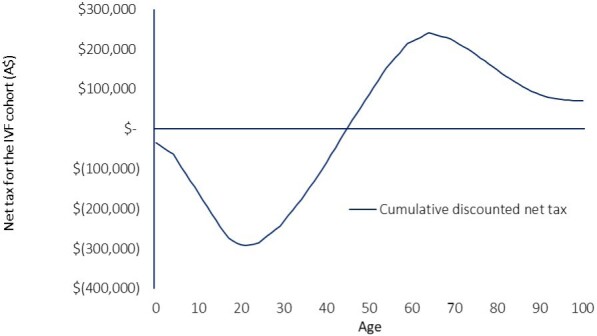
Cumulative Net Discounted Tax Over Lifetime of an ART-Conceived Child Conceived from ART Treatment Performed in 2021 in Australia (Discounted at 3%) Abbreviation: ART, assisted reproduction technology; ICF, in vitro fertilization.

The model generates cumulative discounted net tax contributions at every year of life up to 100 years, adjusted for age-specific mortality. **Figure 1** illustrates the net tax position of an individual at any stage of life. In the early stage of life, a child is a net recipient of government benefits, as reflected by the negative value, with a positive net tax occurring in later years after age 40, as cumulative tax revenues increase relative to transfers. In later years, cumulative net tax decreases as tax payments decrease on average, with increasing reliance on government benefits.

The lifetime taxes and government costs for naturally conceived and ART-conceived children are described in **Table 1**. Lifetime gross taxes per individual were estimated at A$841 631, which consisted of A$580 182 in direct taxation of earnings and A$261 448 in consumption taxes. Lifetime transfer benefits received over the lifetime were estimated at A$744 549. The resulting net tax was estimated at A$97 082. After factoring in those costs required for consumption, the net tax of the ART child was A$70 688. Applying the government costs for a live birth from ART, the fiscal benefit-cost ratio was 2.68.

**Table 1.** Lifetime Cumulative Projections for a Child Naturally Conceived or Conceived Through Assisted Reproduction Discounted at 3% (Australian Dollars)

	**Naturally Conceived (A$)**	**ART-Conceived (A$)**
Earnings		
Lifetime gross income	2 864 051	2 864 051
Taxation		
Direct tax	580 182	580 182
Indirect tax	261 448	261 448
Gross tax	841 631	841 631
Transfer payments		
Government transfers	277 735	277 735
Healthcare costs	292 126	292 126
Educational costs	174 689	174 689
Average cost per live birth		26 394
Total transfers	744 549	744 549
Net tax	97 082	97 082
Outcomes		
Total transfers (-)	-744 549	-744 549
Gross tax (+)	841 631	841 631
Investment (-)		-26 394
Net tax (±)	97 082	70 688
Benefit cost ratio		2.68

Considering the entire birth cohort of 18 364 ART-conceived children from a single year of treatment, the net fiscal gain for government was estimated to be A$1.29 billion over the lifetime of these children.

**Figure 2.**
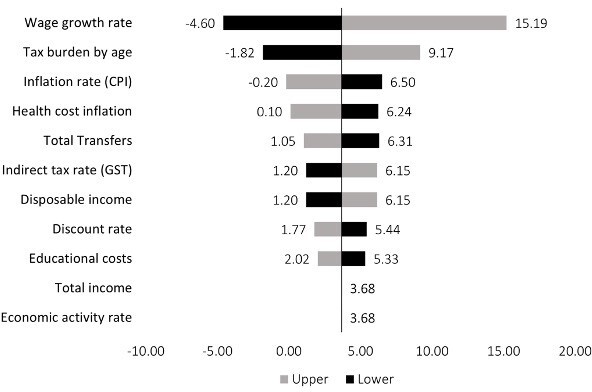
One-Way Sensitivity Analysis Varying All Parameters by ±25% and Influence on Fiscal Benefit Cost Ratio Abbreviations: CPI, consumer price index; GST, Goods and Service Tax.

The sensitivity analysis explored the impact of variation in input variables around the base fBCR of 2.68, as illustrated in **Figure 2**. Varying input parameters illustrates the model is most sensitive to wage growth rates and changes to the tax burden.

## DISCUSSION

The results indicate the Australian government yields important fiscal gains associated with funding infertility services based on projected future net tax revenues of A$1.29 billion from births in a single year attributed to ART. Even with generous welfare benefits paid to Australian citizens, government investments in ART achieve a fBCR of 2.68, meaning that for every A$1 spent in 2021 on ART, the government receives A$2.68 in net present value over the lifetime of the cohort. The decision to fund ART in Australia is based on a range of factors recognizing infertility as a medical condition and pronatalist views. What our analysis shows is that the current policy can also be justified on fiscal grounds.

In Australia, there is a long history of using public economic models, especially the Static Incomes Model (STINMOD), which is linked to population level tax and transfer systems, used to evaluate the long-term impact of government policies.^20,24,37^ As described in this analysis, we have applied a similar modeling approach using age-specific STINMOD data to investigate the cost consequence for the Australian government from investing in ART medical services. The framework described here has been applied previously and published in other settings to inform resource allocation decisions for ART.^22,23^ Furthermore, the framework described here is consistent with the ISPOR (International Society for Pharmacoeconomics and Outcomes Research) guidelines on conducting fiscal analysis of health programs.^19,38^

Many nations around the world are facing aging populations attributed to declining births and increasing life expectancy. While control over individuals’ reproductive choices and longevity are accomplishments to be celebrated, mounting concerns over aging populations are varied and include worker and skill shortages, economic growth prospects, and the inability of government to fund services as the number of workers funding public programs declines.^20^ To support or increase births, governments often turn to family-friendly policies, as well as pronatalist policies that have the explicit aim to increase births (ie, baby bonuses). Evidence supporting these policies are often mixed, offering temporary increases to fertility rates and influencing the timings of births but at considerable expense.^10,39,40^ Recognizing the contribution that ART can make to annual births, reaching 3.5% of national births in Europe with some rates of 5%, governments have started to embrace public funding for fertility treatments as one of the options to address declining births.^41–43^ While many demographers reject the notion that ART can reverse the trend of declining births, it should be seen as part of a policy mix to support families.

In the context of health care, the fiscal modeling framework can be used to evaluate individual health programs and the likely impact on government accounts now and into the future based on investment in healthcare programs.^44^ The applied framework is based on human capital economics, often applied in the evaluation of health programs; the distinction being that a cross-sectorial government perspective is applied that includes tax revenues, financial transfers, and health costs in order to reflect the broader government perspective.^44,45^ This relationship between individuals and government revenue suggests that our work extends beyond the initial concern of evaluating ART investment costs in relation to lifetime fiscal gains for children born from assisted reproduction. This analysis provides a lifetime fiscal projection for an average individual born in Australia; beyond the scope of our work, this framework can be used by other researchers exploring public health interventions in Australia that might alter the fiscal life trajectory of individuals at any age of life. Interested readers can apply these findings to evaluate different government policies similar to what has previously been done for chronic conditions in Australia.^46,47^

It is important to recognize the dynamic nature of the tax transfer system, which has experienced significant evolution over past decades and will continue to evolve. These changes naturally would influence our estimates numerically. Historical trends, however, suggest that policy changes are typically introduced incrementally, thereby exerting minimal impact on the overall effective tax rate. Such an approach suggests a gradual adaptation of the tax and transfer system over a relatively long period, aligning with our assumptions when approximating the long-term fiscal impact. For the fiscal year 2024-2025, it is widely speculated that there will be a nominal reduction in the personal income tax rate of 1.5% to 2.5% for the majority of taxpayers due to recent changes to the tax code in Australia.^48^ Nevertheless, the progressive nature of the income tax system, coupled with projected nominal wage growth, suggests that the real-value impact of these adjustments will likely be more subdued. Thus, despite expected changes in tax and transfer policies, the magnitude of such adjustments is likely to be much smaller than what our estimates’ sensitivity parameters account for, suggesting that these policy shifts are unlikely to fundamentally alter our primary conclusions.

An important consideration when interpreting our results relates to the socioeconomic profiles of those couples likely to seek medical treatment for infertility and how this could influence our fiscal projections. Previous studies have reported that college-educated couples and those with higher household income were more likely to seek fertility care and use more ART.^49,50^ This is likely to be more apparent in countries that require out-of-pocket costs, which could influence the ability to access fertility services. Considering the intergenerational transmission of wealth that exists, meaning that wealthy parents give rise to wealthy children,^51^ this would suggest that the birth cohort from ART would likely earn more in the future. This further suggests that these children may pay higher than average taxes and therefore that our net tax projections are a conservative underestimate of the likely return on investment for the state.

There are several limitations to this modeling approach and policy analysis worth considering when interpreting these findings. First, this analysis does not cover state and local government taxes, such as land tax, council tax, and utility concessions. Additionally, the calculations do not include all in-kind public services such as transportation and security, as well as individual contributions that extend beyond direct financial transactions. While these elements could be considered as part of the broader tax and transfer system, they are excluded due to data limitations and our focus on assessing the direct interactions with the federal tax system, which accounts for over 80% of all tax revenue in Australia.^52^ However, this limitation is acknowledged with the recognition that these components have regional variations that could influence the overall tax and benefit distribution to a limited extent. Second, the analysis reported here is based on the financial relationship between an individual and the state. Our analysis does not account for interactions across economic domains, which can give rise to greater growth due to the likelihood of fiscal multipliers, suggesting that our findings could be underestimates.^53^ Additionally, there are always inherent weaknesses when projecting important parameters over many generations. We have allowed these parameters such as wages and costs to grow based on historical patterns which may not materialize in the future and would clearly impact our results.

Perhaps one of the biggest weaknesses of our work is that we reduce human life to a series of financial transactions between citizens and the state. Our analysis disregards many of the quality-of-life benefits that couples obtain from infertility treatment and those associated with parenthood, which are the main value that fertility treatments deliver to individuals and society.

## CONCLUSION

Infertility is a medical condition with consequences that extend beyond the realm of the healthcare sector, with important economic consequences for government. In this analysis we illustrate in the Australian context that the government benefits in relation to its ongoing commitment to fund assisted reproduction services generating A$2.68 net fiscal gain for every A$1 spent on infertility treatments. Policy changes that might influence the overall cost or the ability of people to access services could influence the underlying projections described in our research.

### Ethics Statement

The analysis described here is a modeling study that projects the fiscal impact of funding policies in Australia for assisted reproduction. There are no interventions or individual subjects recruited for this study, which is based on population wide data for Australia. Because no individual subjects can be identified, therefore, ethics approval was not required.

### Data Availability

The data on which this analysis was based was derived from publicly available data sources, namely, data from the Australian Bureau of Statistics and data from the Australian Tax and Transfer System that is maintained by the National Centre for Social and Economic Modeling at the University of Canberra. Researchers can request access to this data from the relevant data custodian. No proprietary data was used in developing this analysis.

## Supplementary Material

Online Supplementary Material
