# Supplementary material for: Estimating the Fiscal Value of Children Conceived from Assisted Reproduction Technology in Australia Applying a Public Economic Perspective
Source: J Health Econ Outcomes Res. 2025 Apr 18;12(1):148–54. doi: 10.36469/001c.133796 (PMC12009558; doi:10.36469/001c.133796)
Supplement: Online Supplementary Material [file jheor_2025_12_1_133796_279518.pdf]

Journal of Health Economics and Outcomes Research

Online Supplementary Material

Estimating the Fiscal Value of Children Conceived from Assisted Reproduction Technology in Australia Applying a Public Economic Perspective. *JHEOR*. 2025;12(1):148-154. doi:10.36469/jheor.2025.133796

Table S1: Annual Taxes Paid and Disposable Income by Age Groups

Figure S1: Government Spending Per Pupil by Age

Table S2: Educational Expenditure by Australian Government Per Student Over Education Lifetime

This supplementary material has been provided by the authors to give readers additional information about their work.

This is an open-access article distributed under the terms of the Creative Commons Attribution 4.0 International License (CCBY-4.0). View this license's legal deed at http://creativecommons.org/licenses/by/4.0 and legal code at http://creativecommons.org/licenses/by/4.0/legalcode for more information.

Input Data

Age-specific data on income taxes paid and disposable income. The mean annual amount of funds received from federal government including family benefits distributed to eligible children.

Table S1. Government Spending Per Pupil by Age

Age Group, y	Taxes Paid Per Person by Age, Mean (A$)	Disposable Income by Age Including Transfers Allocated to Adults, Mean (A$)	Mean Annual Transfers Received from Government (A$)
0-4			3507
5-9			3308
10-14			3396
15-19	593	9203	3100
20-24	4092	31 468	2819
25-29	9512	45 948	2410
30-34	14 503	56 540	2250
35-39	16 134	60 248	1932
40-44	18 744	64 564	1625
45-49	18 343	62 728	1651
50-54	19 079	62 058	2181
55-59	16 216	56 396	3063
60-64	10 096	46 893	3945
65-69	4573	39 387	9771
70-74	2 30	36 688	13 272
75-79	1604	34 060	15 005
80-84	1678	30 403	16 119
85+	2061	31 778	19 396

Figure S1. Government Spending Per Pupil by Age

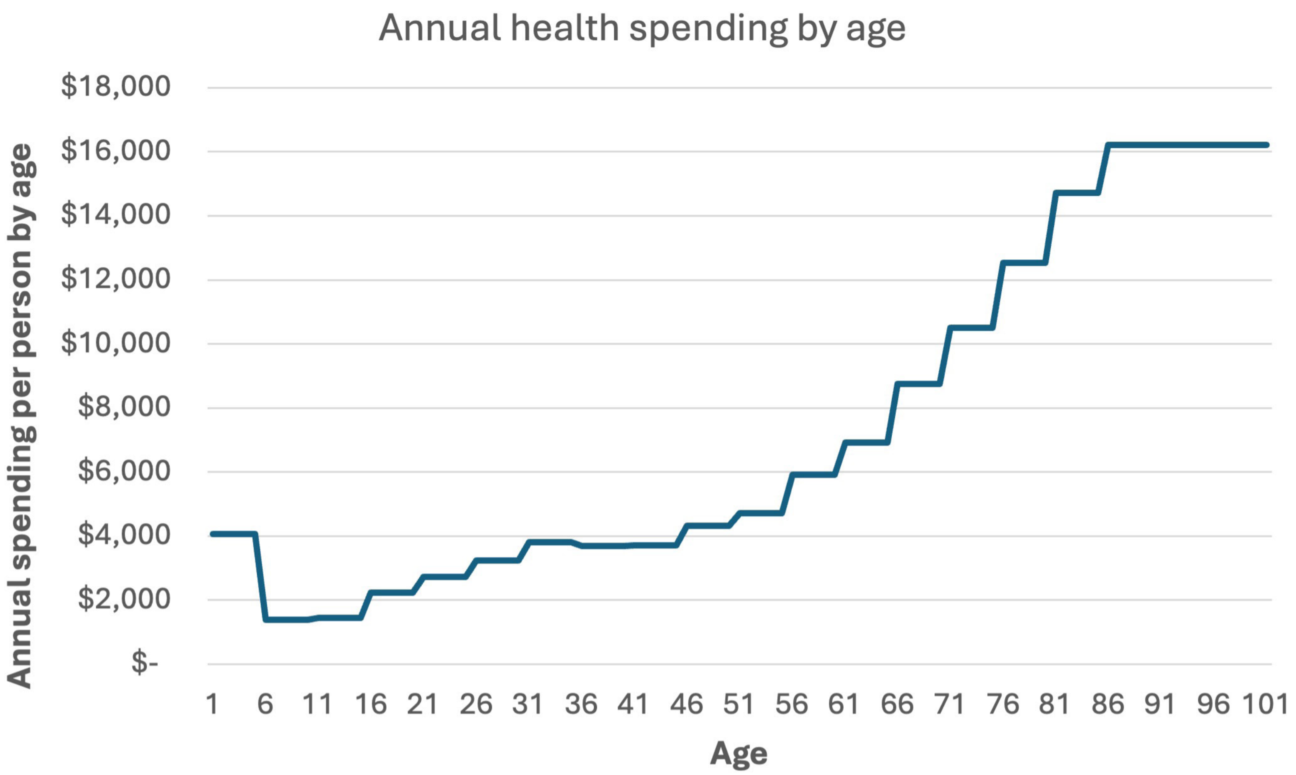

Government spending per pupil was applied to appropriate ages for costs by educational level and adjusting for participation rate.

Table S2. Educational Expenditure by Australian Government Per Student Over Education Lifetime

Education Level	Attainment, %[23]	Cost Per Pupil Per Year (A$)[24]	Weighted Cost Per Person (A$)
Primary education	100.0	12 311.00	12 311
Secondary education	100.0	13 841.00	13 841
Upper secondary education	90.1	13 841.00	12 470
Tertiary	44.6	11 303.00	5041

Sensitivity Analysis

The parameters of discount rate, disposable income, annual income, health cost inflation rate, tax burden, wage growth rate, government transfers, healthcare costs, inflation rate, educational costs, Goods and Service Tax rate, economic activity rate, and IVF treatment costs were all varied by ±25% in the 1-way sensitivity analysis.
